# Direct Activation of Ribosome-Associated Double-Stranded RNA-Dependent Protein Kinase (PKR) by Deoxynivalenol, Anisomycin and Ricin: A New Model for Ribotoxic Stress Response Induction

**DOI:** 10.3390/toxins6123406

**Published:** 2014-12-16

**Authors:** Hui-Ren Zhou, Kaiyu He, Jeff Landgraf, Xiao Pan, James J. Pestka

**Affiliations:** 1 Department of Food Science and Human Nutrition, Michigan State University, East Lansing, MI 48824, USA; E-Mail: zhouh@msu.edu; 2 Department of Microbiology and Molecular Genetics, Michigan State University, East Lansing, MI 48824, USA; E-Mail: hekaiyu@gmail.com; 3 Research Technology Support Facility, Michigan State University, East Lansing, MI 48824, USA; E-Mail: landgra1@msu.edu; 4 Center for Integrative Toxicology, Michigan State University, East Lansing, MI 48824, USA; E-Mail: daxiaopenny@gmail.com; 5 Department of Biochemistry and Molecular Biology, Michigan State University, East Lansing, MI 48824, USA

**Keywords:** PKR, ribotoxic stress, rRNA, deoxynivalenol, anisomycin, ricin, translational inhibitor, quantitiative Western analysis, RNA immunoprecipitation

## Abstract

Double-stranded RNA (dsRNA)-activated protein kinase (PKR) is a critical upstream mediator of the ribotoxic stress response (RSR) to the trichothecene deoxynivalenol (DON) and other translational inhibitors. Here, we employed HeLa cell lysates to: (1) characterize PKR’s interactions with the ribosome and ribosomal RNA (rRNA); (2) demonstrate cell-free activation of ribosomal-associated PKR and (3) integrate these findings in a unified model for RSR. Robust PKR-dependent RSR was initially confirmed in intact cells. PKR basally associated with 40S, 60S, 80S and polysome fractions at molar ratios of 7, 2, 23 and 3, respectively. Treatment of ATP-containing HeLa lysates with DON or the ribotoxins anisomycin and ricin concentration-dependently elicited phosphorylation of PKR and its substrate eIF2α. These phosphorylations could be blocked by PKR inhibitors. rRNA immunoprecipitation (RNA-IP) of HeLa lysates with PKR-specific antibody and sequencing revealed that in the presence of DON or not, the kinase associated with numerous discrete sites on both the 18S and 28S rRNA molecules, a number of which contained double-stranded hairpins. These findings are consistent with a sentinel model whereby multiple PKR molecules basally associate with the ribosome positioning them to respond to ribotoxin-induced alterations in rRNA structure by dimerizing, autoactivating and, ultimately, evoking RSR.

## 1. Introduction

Many toxins produced by microbes and plants inhibit translation by binding to the ribosome and interfering with initiation, elongation and/or termination [1]. Besides translation inhibition, these biotoxins are capable of activating mitogen-activated protein kinases (MAPKs) via a process known as the ribotoxic stress response (RSR) [2,3,4,5]. RSR is of critical importance because it drives aberrant gene expression and apoptosis that are downstream hallmarks of ribotoxin-induced pathophysiologic effects. It has been proposed that the RSR involves damage to the 3' end of the large 28S ribosomal RNA (rRNA) molecule, which functions in aminoacyl-tRNA binding, peptidyl transferase activity and ribosomal translocation [2]. However, the mechanisms by which this or other ribosomal damage are sensed and communicated to downstream stress signaling kinases remain poorly understood.

The prototypical ribotoxin deoxynivalenol (DON), a trichothecene mycotoxin, triggers activation of p38 and c-Jun *N*-terminal kinase (JNK) MAPKs with robust downstream sequelae including cytokine and chemokine gene expression as well as programmed cell death in leukocytes [6,7]. Cell culture studies on the effects of DON and other ribotoxins strongly indicate that double-stranded RNA-activated protein kinase (PKR) is a critical upstream sensor and transducer of RSR [7,8,9,10].

PKR is a constitutively-expressed serine/threonine protein kinase known to associate with the ribosome [11,12,13]. Activated PKR was initially found to phosphorylate eukaryotic initiation factor 2α (eIF2α) at serine 51, which increases its affinity for the (guanine nucleotide exchange factor) GTP exchange factor eIF2β thereby inactivating the complex, and*,* as a result, inhibiting translation [14]. Based on these findings, PKR is postulated to play role in the antiviral response to dsRNA-containing viruses. Besides translational inhibition, PKR can also activate a wide range of factors including signal transducer and activator of transcription (STAT), interferon regulatory factor 1 (IRF-1), p53, JNK, p38 and NF-κB [13,15,16] that play central modulatory roles in gene expression, cell growth, tumor suppression, and apoptosis [17,18,19].

PKR is rapidly activated by DON in murine RAW 264.7 macrophages and human U-937 monocytes, as evidenced by its autophosphorylation and the subsequent phosphorylation of its downstream substrate eIF2α [7]. Identical findings were made for the ribotoxins anisomycin and emetine. PKR inhibitors suppress DON-induced MAPK activation as well as expression of cytokines and chemokines, indicating that this kinase plays a critical role in RSR [7,10,20,21]. In addition, DON, anisomycin, and emetine evoke caspase-3 activation and DNA fragmentation in wild type but not in PKR-deficient U937 cells, suggesting that PKR is required not only for initiation of RSR, but also for ribotoxin-driven apoptosis [7]. While it is apparent that DON and other ribotoxins activate PKR and trigger downstream RSR-associated MAPK signaling pathways capable of regulating gene expression and apoptosis, the upstream mechanisms are still unclear.

The eukaryotic 80S ribosome is composed of a 40S subunit consisting of a single 18S rRNA molecule and 33 proteins, and a 60S subunit consisting of 3 rRNA molecules (5S, 5.6S and 28S) and 46 proteins [22]. When expression of PKR protein was studied in a yeast model using density gradient centrifugation in conjunction with immunoblotting, over 70% of the kinase was found to fractionate with the 40S and 60S subunits and 80S particles of the ribosome [23]. Similar findings have been made in human U-937 monocytes [24]. PKR has also been linked to the rapid activation of hematopoietic cell kinase (Hck), p38 and ERK within the ribosomal compartment of DON-treated mononuclear phagocytes [9]. Furthermore, DON recruits p38 to the ribosome in wild-type but not PKR-deficient peritoneal macrophages suggesting that ribosome-associated PKR is essential for DON-induced p38 activation.

PKR contains two double-stranded (ds)RNA binding domains (DRBDs) and one kinase domain whose activity is self-inhibited by PKR binding of the DRBDs in an intramolecular manner [25,26,27,28]. In a widely accepted model of activation, inactive monomers of PKR dimerize after associating with dsRNAs in close proximity, thereby resulting in their autophosphorylation and self-activation. Most RNA consists of a single strand that can fold back on itself to form more complex structures [29]. Central to these structures are hairpins that are comprised of both a double-stranded stem with Watson-Crick base pairing and a loop in which the backbone changes directionality. PKR DRBDs bind to dsRNA in a sequence-independent manner [11]. It has been previously established that PKR requires binding to dsRNA sequences longer than 30 nts for its dimerization and autophosphorylation [30]. The dependence of PKR-ribosome association on both DRBDs [23,31] implies that this kinase likely interacts to a large extent with ribosomal RNA (rRNA).

At least two possible models can be envisioned for ribotoxin-induced PKR activation. One possibility is a sentinel model in which PKR monomers basally associate with the ribosome and rRNA. Upon interaction with a ribotoxin, one or more portions of rRNA reposition and thereby promote dimerization of the PKR monomers followed by autophosphorylation and self-activation. A second possibility is a sequential mode whereby a ribotoxin first associates with rRNA, inflicting damage and/or altering its structure thereby exposing new double-stranded (ds)rRNA regions. This could sequentially elicit (1) binding of two or more PKR monomers in close proximity to the damaged site; (2) dimerization of these monomers and finally; (3) autophosphorylation and self-activation of the kinase. The purpose of this investigation was to further characterize the PKR’s interactions with the ribosome and rRNA and relate these findings to activation of the kinase. The results presented herein provide new supportive evidence that favors the sentinel model of ribotoxin-induced PKR activation.

## 2. Results

### 2.1. DON Induces PKR-Dependent MAPK Phosphorylation in HeLa Cells

**Figure 1:**
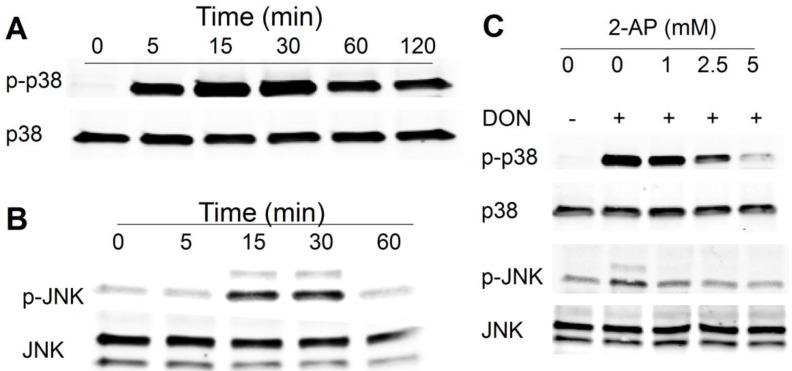
Trichothecene deoxynivalenol (DON) induces protein kinase (PKR)-dependent ribotoxic stress response in HeLa cells. Kinetics of DON-induced phosphorylation of (**A**) p38; and (**B**) c-jun N-terminal kinase(JNK) HeLa cells were treated with 500 ng/mL of DON for indicated time intervals and then p38 and JNK phosphorylation was determined by Western analysis; (**C**) PKR inhibitor 2-aminopurine (2-AP) suppresses DON-induced p38 and JNK phosphorylation. HeLa cells were pretreated with PKR inhibitor 2-AP at indicated concentrations for 1 h, exposed to DON (500 ng/mL) for 15 min and then p38 and JNK phosphorylation was determined by Western analysis. Data are representative of at least two replicate experiments.

Western blotting revealed that DON (500 ng/mL) induced robust p38 phosphorylation in 5 min in HeLa cells, which was maximal at 15 min and lasted up to at least 2 h (Figure 1A). DON also evoked transient phosphorylation of JNK at 15 and 30 min (Figure 1B). The PKR inhibitor 2-AP concentration-dependently suppressed the p38 and JNK (Figure 1C). These findings recapitulated prior findings in mononuclear phagocytes that DON induces RSR in a PKR-dependent manner, indicating that HeLa cells are appropriate for the investigation of PKR-ribosome interactions.

### 2.2. Multiple PKR Molecules Basally Associate with the 40S, 60S, 80S and Polysomal Fractions of the Ribosome in HeLa Lysate

**Figure 2:**
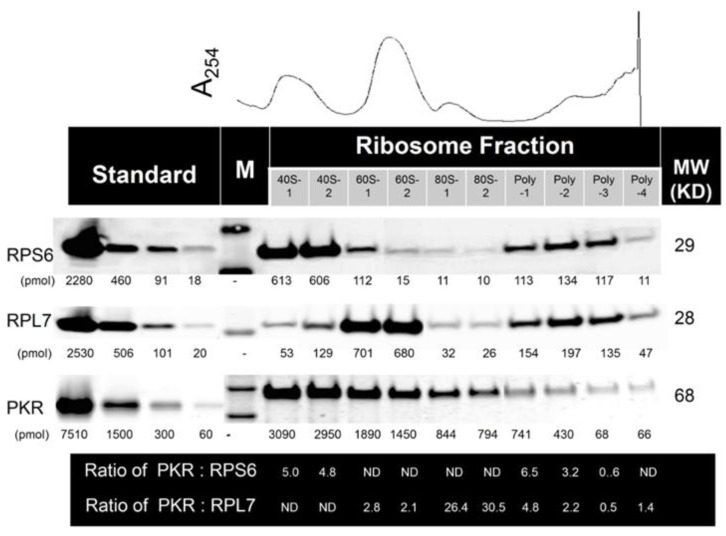
Multiple PKR molecules associate with 40S, 60S, 80S and polysomes in naïve HeLa cells. Stoichiometry was assessed by density gradient fractionation of ribosomes from naive HeLa cell lysate in conjunction with quantitative Western blotting using the Li-Cor Odyssey Infrared Imaging System. M indicates molecular weight markers. ND indicates not determined; Results are representative of three identical.

The relative ratios of PKR to ribosome fractions were estimated by employing density gradient fractionation of ribosomes from HeLa cell lysate in conjunction with quantitative Western blotting (Figure 2). When data were averaged from three independent experiments, the molar ratios (mean ± SEM) of PKR to 40S, 60S, 80S and polysome were estimated to be 6.7 ± 0.9, 2.3 ± 0.2, 23.4 ± 2.9 and 2.6 ± 0.6, respectively. These observations suggest that multiple molecules of PKR basally associated with individual ribosomal subunits and particles in HeLa cell lysates.

**Figure 3:**
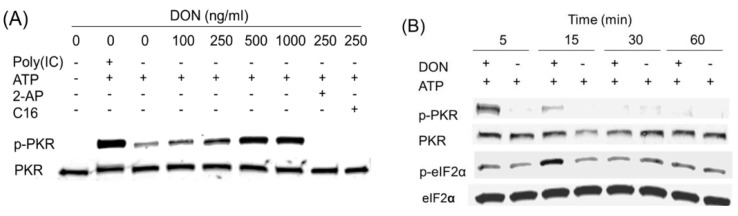
DON induces transient PKR activation in HeLa-based cell-free system. (**A**) DON (100, 250, 1000 ng/mL), poly (IC) (100 ng/mL) and/or PKR inhibitors, 2-AP (2 mM) and C16 (2 µM), were added to a cell-free system comprised of HeLa-based cell-derived, translationally active cell-free system containing ribosomes and ATP but devoid of cell membrane, nuclei, mitochondria, DNA and mRNA. After incubation for 20 min at 30 °C, Western analysis was conducted with PKR and p-PKR antibodies; (**B**) HeLa-based cell-free assay mixtures were incubated with DON (250 ng/mL) for indicated time intervals and subjected to Western blotting with PKR, p-PKR and eIF2α, *p*-eIF2α antibodies. Data are representative of at least three replicate experiments.

### 2.3. DON Induces PKR Activation in ATP-Containing HeLa Lysate

DON’s effects on PKR phosphorylation were characterized in a translationally active cell-free system that employed HeLa cell extract that was devoid of nuclei, mitochondria, lipids, DNA and mRNA. Exogenous addition of ATP, the phosho-group donor, induced modest basal PKR phosphorylation at 0.1 mM, however, this response was greatly elevated by inclusion of the prototypical PKR activator poly (IC) or DON at 100 to 1000 ng/mL in concentration-dependent manner (Figure 3A). These responses were markedly suppressed by the PKR inhibitors 2-AP and C16 suggesting that PKR phosphorylation resulted from autophosphorylation. DON at 250 ng/mL was found here to transiently activate PKR as early as 5 min, and this was attenuated at 15 min (Figure 3B). PKR autoactivation is known to induce eIF2α phosphorylation at serine 51 [14]. Consistent with this, eIF2α was phosphorylated marginally at 5 min and maximally at 15 min. Accordingly, the HeLa extract assay system contained all the necessary functional components to reconstitute DON-induced PKR activation and eIF2α phosphorylation under cell-free conditions.

**Figure 4:**
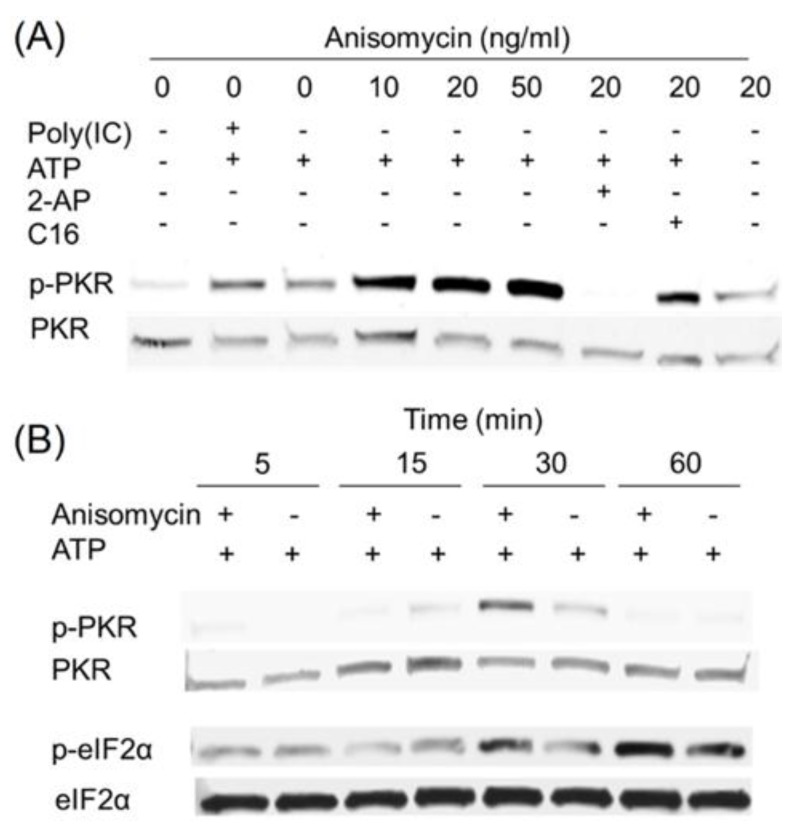
Anisomycin induces transient PKR activation in HeLa cell-free system. (**A**) Anisomycin (10 to 50 ng/mL), poly (IC) (100 ng/mL) and/or PKR inhibitors, 2-AP (2 mM) and C16 (2 µM), were added to HeLa-based cell-free assay mixtures. After incubation for 20 min at 30 °C, Western analysis was conducted with PKR and p-PKR antibodies; (**B**) HeLa-based cell-free assay mixtures were incubated with anisomycin (20 ng/mL) for indicated time intervals and subjected to Western blotting analysis with PKR, p-PKR and eIF2α, p-eIF2α antibodies. Data are representative of at least three replicate experiments.

### 2.4. Anisomycin and Ricin Induce PKR Activation in HeLa Lysate

**Figure 5:**
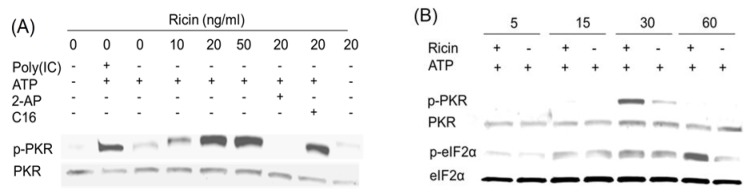
Ricin induces transient PKR activation in HeLa cell-free system. Experiment conditions were as described in Figure 4 legend except that ricin was used instead of anisomycin.

Inclusion of the ribotoxins anisomycin (Figure 4A) or ricin (Figure 5A) at 10 to 50 ng/mL in ATP-containing HeLa-lysates also induced PKR phosphorylation in a concentration-dependent fashion. As observed for DON, these responses were markedly suppressed by the PKR inhibitors 2-AP and C16. When the kinetics of PKR phosphorylation were measured, anisomycin (Figure 4B) and ricin (Figure 5B) were found to activate PKR at 30 min. Subsequent eIF2α phosphorylation was also detected at 30 and/or 60 min. Therefore, like DON, other ribotoxins were capable of activating PKR in this HeLa-based cell-free model.

**Figure 6:**
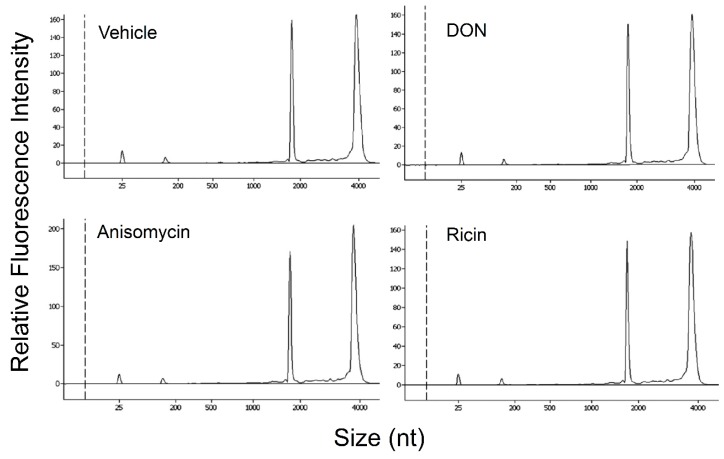
Ribotoxins do not affect rRNA integrity prior to PKR activation in HeLa-based cell-free assay mixtures. HeLa-based cell-free assay mixtures were incubated with vehicle, DON, anisomycin (20 ng/mL) or ricin (20 ng/mL) for 30 min at 30 °C and then subjected to RNA purification and capillary electrophoresis. Data are representative of at least three replicate experiments.

### 2.5. DON, Anisomycin and Ricin Do Not Affect rRNA Integrity in HeLa Lysate

To assess whether rRNA cleavage occurs prior to or during PKR activation, rRNA integrity in HeLa assay mixtures was measured after incubation in the presence and absence of 250 ng/mL DON for 30 min. Capillary electrophoresis revealed that the rRNA integrities were identical following either treatment (Figure 6). Similarly, anisomycin (20 ng/mL) and ricin (20 ng/mL) exposure had no effect on rRNA over this same time period. Thus, under conditions where PKR activation was observed, DON or the other ribotoxins did not induce RNA cleavage in the HeLa extract.

**Figure 7:**
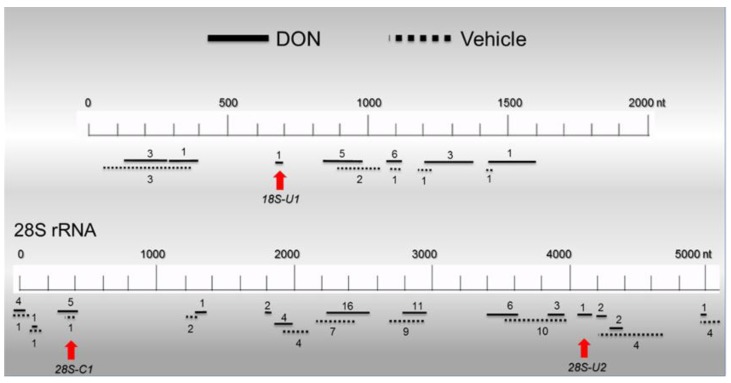
Distribution of RNA-IP-identified PKR-associated sequences in HeLa-based cell-free assay mixtures. HeLa lysate assay mixtures were incubated with or without DON (250 ng/mL) at 30 °C for 20 min and subjected to RNA-IP using PKR-specific antibody in four independent experiments. Following purification, cloning and sequencing of immunoprecipitated PKR-associated rRNAs, sequences were aligned to human 18S (NCBI Reference Sequence: NR-003286.2) and 28S (NCBI Reference Sequence: NR_03287.2) ribosomal RNA genes and their relative sizes and locations depicted above. The upper (solid) and lower (dotted) lines indicate the PKR-associated sequences identified from DON-treated and control, respectively. Integers in parentheses denote the number of clones recovered that were recovered in designated region.

### 2.6. Basal PKR Association with rRNA in HeLa Lysates Is Unaffected by DON

**Figure 8:**
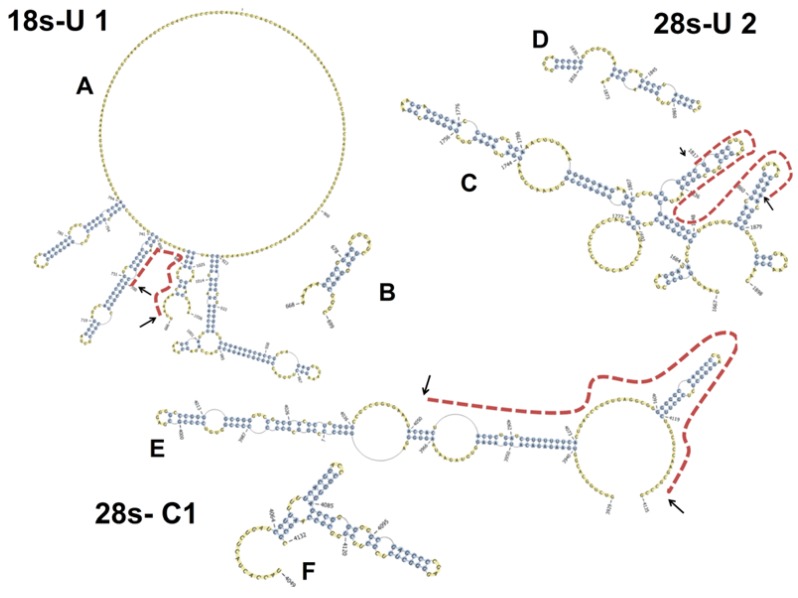
Predicted secondary structures of rRNAs recovered by PKR-specific RNA-IP from HeLa-based cell-free assay mixtures contain significant double-stranded regions. Representative PKR-bound rRNA sequences ((**A**) 18S-U1; (**C**) 28S-U2; and (**E**) 28S-C1) were mapped and aligned to human 18S and 28S rRNAs and then depicted with PseudoViewer. Dashed lines indicate regions of dsrRNA available for interaction with PKR DRBD based on predicted secondary structure of the fragment. In addition, secondary structures of the isolated PKR-associated rRNA fragments ((**B**) 18S-U1; (**D**) 28S-U2; and (**F**) 28S-C1) were predicted to confirm double-stranded regions using PknotsRG with minimum free energy and then depicted with PseudoViewer.

We assessed the potential for PKR to interact with RNA in the lysate and further determined whether this was impacted by the presence of DON. This was achieved by RNA immunoprecipitation with PKR-specific antibody of both vehicle- and DON-treated HeLa assay mixtures. Recoveries (mean ± SEM) in four independent experiments were 6.64 ± 0.25 and 6.28 ± 0.28 ng RNA/mg antibody protein for vehicle- and DON-treated assay mixtures, respectively. rRNA recoveries for PKR-specific antibody were 4 to 5 times higher than those for an equivalent amount of isotype-matched control antibody (data not shown). Accordingly, these results suggest that most of the RNAs recovered with the PKR antibody were specifically associated with the kinase, and furthermore, DON treatment did not significantly alter the amount of RNA bound to PKR. To characterize potential PKR binding sites in rRNA, RNAs recovered from vehicle- and DON-treated assay mixtures by PKR-specific RNA-IP were cloned and sequenced (Figure 7). Numbers and locations of recovered PKR-associated sequences were largely similar for vehicle-treated (51 clones) and DON-treated (79 clones) assay mixtures and included sites on both 18S (80–350, 668–699, 820–930 nts) and 28S (0–430 nts, 1816–1873, 2370–2620, 2790–2960, 3590–3970, 4049–4132, 4200–4750 nts) rRNA molecules (Figure 7). Significant regions of dsrRNA with hairpins were observed when representative hot spots were mapped to either secondary structures previously reported for intact 18S and 28S rRNA molecules (Figure 8A,C,E) or to predicted secondary structures of the isolated fragments, (Figure 8B,D,F).

**Figure 9:**
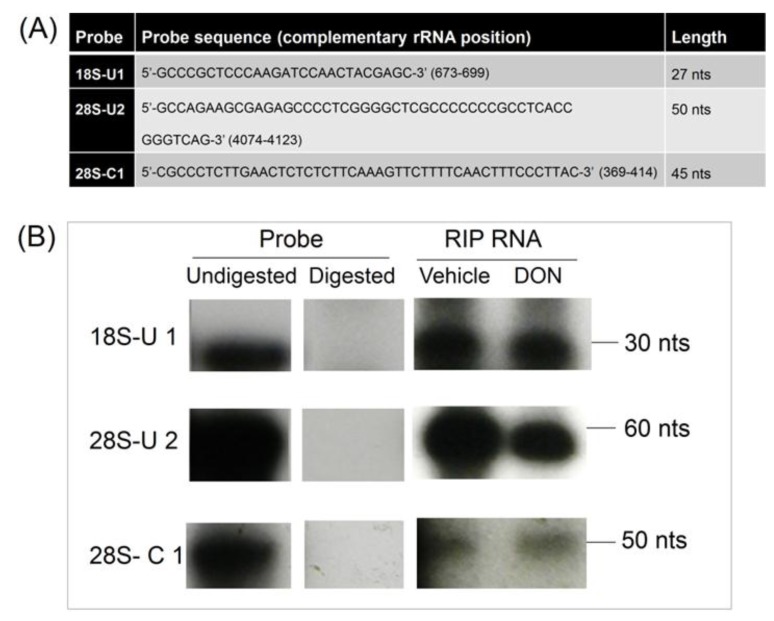
DON does not alter PKR binding to rRNA in HeLa-based cell-free assay mixtures. (**A**) Sequences and sizes of 32P labeled probes for 18S-U1 28S-U2 and 28S-C1; (**B**) Radiolabeled probes were incubated with immunoprecipitated RNAs from control and DON-treated HeLa extracts, subjected to RNase treatment, separated by urea-PAGE and analyzed by autoradiography.

RNase protection assays were performed using specific radiolabeled probes within the above-described sequences 18S-U1 28S-U2 and 28S-C1 RNase protection assays were performed by incubating with ^32^P labeled probes for 18S-U1, 28S-U2 and 28S-C1 and with RNA-IP RNA from control and DON-treated HeLa extracts. Resultant RNAs were subjected to RNAse treatment, denatured, separated by 15% urea-PAGE and then were analyzed by autoradiography. Consistent with the rRNA recovery data following PKR-specific RNA-IP, the kinase appeared to similarly bind to these sites regardless of whether they were pretreated with vehicle or DON (Figure 9). Accordingly, these results suggest that PKR associated with rRNA in the HeLa lysate, independently of the presence of DON.

## 3. Discussion

**Figure 10:**
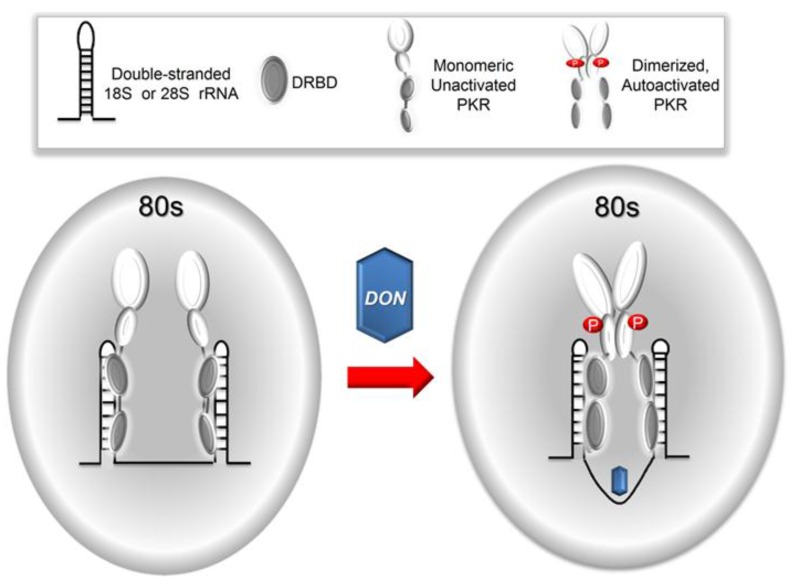
Hypothetical sentinel model for DON-induced PKR activation. As depicted below, our data favor a sentinel model in which multiple copies of PKR associate with ribosomes. Consistent with established models for PKR activation [25,26,27,28], monomers of this kinase might be well-positioned to rapidly sense subtle perturbations in rRNA secondary and/or tertiary structure induced by association with DON or other ribotoxins. Such changes in rRNA structure could reposition a PKR monomer in close proximity to another monomer thereby causing dimerization. The latter could evoke autophosphorylation, PKR activation and, ultimately, initiate RSR.

Prior studies in DON-treated mononuclear phagocytes suggest that PKR functions as a universal sensor/transducer for early RSR as reflected by MAPK activation [7]. The HeLa cell is another culture model that has been previously employed to classify translational inhibitors [1] and has been used to study RSR [32]. Our demonstration that DON induced PKR-mediated MAPK activation in HeLa cultures confirms that this cell line was applicable to mechanistic investigation of DON-induced PKR activation. The observations described herein are novel for several reasons. First, this report precisely quantitates the molar ratios of PKR to ribosomal subunits and particles. These stoichiometry data suggest that in HeLa cells, multiple PKRs basally associate with the ribosome subunits and particles, most notably the monosome. Second, the data presented here demonstrate that ribotoxin-induced PKR activation could be reconstituted in a cell-free system. Third, characterization of sites of PKR association with rRNA in this HeLa lysate model by RNA-IP revealed that, consistent with stoichiometric data, multiple copies of PKR basally associate with 18S and 28S rRNA. Importantly, the association of PKR with rRNA was not further impacted by the presence of the ribotoxin DON. In further support of this finding, it was previously determined that although DON induces phosphorylation of ribosome-associated PKR, it did not qualitatively affect the amount of PKR associated with the ribosome [9]. Taken together, these data are more consistent with a sentinel model (Figure 10) for ribotoxin-induced PKR activation than a sequential model.

The stoichiometry studies illustrate that while 7 and 2 PKR molecules associated with the 40S and 60S subunits, respectively, the molar ratio was increased to 23 in the 80S monosome. This implies that potential rRNA binding sites for PKR’s two DRBDs might be greatly magnified when the 40S and 60S subunits are complexed to form the 80S monosome. Accessible binding sites for PKR might be synergistically increased by the interaction of 18S and 28S rRNAs in the monosome within the context of ribosomal proteins. Interestingly, the PKR:monosome ratio decreased to 3 in the polysome fraction indicating that these potential PKR binding sites are significantly reduced in actively translating ribosomes. Based on these stoichiometric data, it might be predicted that the monosome is particularly well-suited to launch the RSR.

A critical and unique aspect of this stoichiometry study was the usage of infrared fluorophores [33]. This approach is not only more sensitive than chemiluminescence but allows a wider dynamic range (2 or more orders of magnitude) than the latter method. Another key consideration is the protocol for ribosome fractionation. The methods for ribosome isolation developed in the 1960s and 1970s are still largely employed with relatively minor modifications. These involve differential or density gradient ultracentrifugation of cell lysates to yield ribosomal subunits and particles. Importantly, protein–ribosome interactions have a range of salt sensitivities. While high stringency salt conditions could elicit dissociation of specific protein-ribosome complexes, lower stringency might allow relatively non-specific proteins to remain associated with the ribosome thus eliciting a higher background signal. Based on our recent study of the ribosome interactome [34], we chose moderate salt conditions for PKR stoichiometry measurements. Finally, it should be emphasized that we did not rely on manufacturer label concentrations for preparing our PKR, RPS6 and RPL6 standards. These might be inaccurate depending on the method used by the manufacturer to estimate protein content which is typically based on the responses of a standard protein such as bovine serum albumin. Notably classical colorimetric methods can yield different estimates among proteins because of differences in amino acid sequence, isoelectric point, secondary structure, and the presence of certain side chains or prosthetic groups. To avoid these problems, we used molar extinction coefficients determined from specific sequence of each standard peptide which are based on the spectral contributions of the Tyr, Trp and Cys at A280 [33,35].

HeLa lysates possess several advantages over intact cells for studying the relationship between rRNA and PKR activation. First, intact cells normally contain 2 to 5 percent dsRNA in native heterogeneous nuclear ribonucleoproteins [36,37], which could potentially translocate to cytoplasm and activate PKR upon toxin treatment. Second, cytoplasmic poly (A)-rich RNA reportedly activates PKR *in vitro* and *in vivo* [38,39,40,41]. Since the HeLa lysate does not contain these cellular RNAs, it reduces the possibility of artifactual activation of PKR by them. Thus, the demonstration that DON could activate PKR in the HeLa-based lysate is consistent with a role for rRNA in the autophosphorylation/activation of this kinase and ultimately, initiation of RSR. The findings that the ribotoxins anisomycin and ricin could also activate PKR in the HeLa-based cell-free preparation indicate that this reconstituted model is sufficiently robust to serve as a general model to study the early stages of RSR.

A key finding of this investigation was that PKR molecules similarly associated with 18S and 28S rRNA sequences in a region-specific manner, regardless of the presence and absence of the DON. Although dsRNA is known to associate with PKR DRBDs in a sequence-independent manner [11], kinase access to 18S and 28S rRNA is likely to be restricted to freely available hairpin structures, *i.e.*, those dsrRNA regions not already interacting with other rRNA or one of the 79 core ribosomal proteins [22,42,43]. Of special significance to the present investigation, a previous study reported that ribosome-associated PKR is monomeric [44] suggesting that the kinase associates with 18S and 28S rRNAs in its monomeric form. DON and other ribotoxins might alter the conformation of some of these rRNAs sufficiently to allow PKR monomers to reposition in closer proximity to other monomers. This would enable them to respond by sequentially dimerizing, autoactivating and, finally, initiating RSR. In further support of this contention, DON appears to bind to 40S and 60S subunits as well as 80S particles [24]. Accordingly, these findings suggest that DON and other ribotoxins could potentially activate ribosome-associated PKR molecules by disturbing 18S and 28S rRNA secondary and/or tertiary structures.

We have previously demonstrated in RAW 264.7 cells that DON induces late stage (3 to 6 h) caspase-dependent rRNA cleavage at multiple sites in 18S and 28S RNAs [45,46]. It is thus theoretically possible that ribotoxin exposure might also initiate early rRNA fragmentation, which could expose short pieces of dsrRNA that would be capable of activating PKR. Our integrity data, coupled with kinetic studies, however, suggest that PKR activation was not preceded by rRNA cleavage in ATP-containing HeLa cell lysates.

## 4. Experimental Section

### 4.1. Assessment of PKR-Dependent MAPK Activation in HeLa Cells

HeLa cells (ATCC, Rockville, MD, USA) were cultured in Dulbecco’s modified Eagle’s medium (DMEM) supplemented with 10% (*v*/*v*) fetal bovine serum (Atlanta Biologicals, Lawrenceville, GA, USA), streptomycin (100 μg/mL) and penicillin (100 U/mL) at 37 °C in a humidified atmosphere with 5% CO_2_. The capacity of DON to rapidly elicit RSR in this cell line was confirmed as previously described. DON (500 ng/mL) was incubated with the HeLa cells (10^6^/mL) for various time intervals up to 120 min. Cells were analyzed for p38 and JNK phosphorylation by Western analysis using a Li-Cod Odyssey Infrared Imaging System (Lincoln, NE, USA) as previously described [24]. PKR involvement in DON-induced p38 and JNK phosphorylation was verified by 30 min preincubation with the specific inhibitor 2-aminopurine (Sigma-Aldrich, St. Louis, MO, USA) at concentrations ranging from 1 to 5 mM prior to DON incubation and Western analysis.

### 4.2. Quantitation of PKR: Ribosome Stoichiometry in HeLa Cells

Stoichiometric ratios of PKR to ribosomal 40S, 60S, 80S and polysomal fractions in HeLa cell lysates were determined by density gradient centrifugation in conjunction with quantitative Western blotting. Cells were washed twice with ice-cold phosphate-buffered saline (PBS) and lysed in 500 μL ice-cold polysome extraction buffer (PEB) consisting of 50 mM KCl, 10 mM MgCl_2_, 15 mM Tris–HCl (pH 7.4), 1% (*v*/*v*) Triton X-100, 0.1 mg/mL cycloheximide, 1 mM DTT, protease inhibitor (Sigma, St. Louis, MO, USA), and phosphatase inhibitor (Santa Cruz Biotechnology, Santa Cruz, CA, USA) [34]. Cell lysates were centrifuged at 16,000× *g*, 4 °C, for 15 min to remove nuclei, mitochondria and cell debris.

The resultant ribosome-containing extract (4 mL) was layered on a 28 mL linear sucrose gradient solution (10%–50% [*w*/*v*] sucrose containing 50 mM KCl, 10 mM MgCl_2_, 15 mM Tris-HCl (pH 7.4), 0.1 mg/mL cycloheximide and protease inhibitor) that was prepared in 36 mL Sorvall centrifuge tubes with an ISCO 160 Gradient Former (Teledyne ISCO, Lincoln, NE, USA). Following centrifugation at 28,000× *g* for 16 h at 4 °C, 40S, 60S, 80S and polysome fractions were isolated from the gradient at a rate of 1 mL per min into 2 mL tube by upward displacement using an ISCO Density Gradient Fraction Collector, consisting of a needle-piercing device with a syringe pump connected to an EM-1 UV monitor for continuous measurement of the absorbance at 254 nm. Proteins from each fraction were precipitated by slow addition of trichloroacetic acid to a final concentration of 10% (*w*/*v*) followed by overnight incubation at 4 °C. Pellets were recovered by centrifugation (10,000× *g* for 15 min), washed with cold acetone twice, and air dried. Proteins were suspended in 8 M urea, 10 mM HEPES, pH 8.0, and protein content was determined by BCA Protein Assay (Pierce, Rockford, IL, USA).

Quantitative Western analysis was based on a previously published protocol [47]. Protein standards used were RPS6 (residues 1–249 [249 aa], *N*-terminal His tag, MW 29 kD), RPL7 (residues 17–248 [232 aa], *N*-terminal His tag, MW 28 kD) (Abcam, Cambridge, MA, USA) and PKR (residues 252–551 [300 aa], *N*-terminal GST tag, MW 62 kD) (Life Technologies, Grand Island, NY, USA). Proteins were reconstituted and concentrations determined with a NanoDrop 2000 UV-Vis Spectrophotometer (ThermoFisher, Wilmington, DE, USA) employing the molecular extinction coefficients at A_280_ (cm^−1^ M^−1^) for each specific peptide (RPL6 = 12450, RPL7 = 25580, PKR = 71630). Extinction coefficients were determined by entering sequence data into the Peptide Property Calculator (http://www.basic.northwestern.edu/biotools/proteincalc.html, last accessed on 12/10/2014) [33,35]. Gradient samples or standards were separated on 4%–20% Mini-Protean TGX Precast Gel (Bio-Rad, Hercules, CA, USA) and then transferred to an Immobilon-FL membrane (Millipore, Billerica, MA, USA). Following incubation with blocking buffer (Li-Cor) for 1 h at 25 °C, membranes were further incubated overnight at 4 °C with specific antibodies for RPL7, RPS6 (Bethyl Labs, Montgomery, TX, USA) and PKR (Santa Cruz, Santa Cruz, CA, USA) diluted in blocking buffer. Blots were washed three times for 10 min with 50 mM Tris-HCl, 150 mM NaCl, 0.1% (*v*/*v*) Tween 20, (pH 7.5), and then incubated with either secondary IRDye 680 goat anti-rabbit or IRDye 800CW goat anti-mouse IgG antibodies (Li-Cor) diluted in blocking buffer for 1 h at 25 °C. After washing three times, infrared fluorescence intensities of the resultant blots were measured on an Odyssey Infrared Imaging System using Li-Cor software v.3.0. (LI-COR Biosciences, Lincoln, NE, USA) RPL7, RPS6 and PKR concentrations in each ribosomal fraction were determined by power regression analysis of standard curves plotting log of integrated fluorescence intensity *vs.* log concentration. Data were pooled from three independent experiments to calculate ratios of PKR:40S (using RPL6 as reference protein) and PKR:60S, PKR:80S and PKR:polysome (using RPL7 as reference protein).

### 4.3. Ribotoxin-Induced PKR Activation in Translationally-Active Cell-Free HeLa Lysates

The effects of DON, anisomycin (Sigma-Aldrich) and ricin (Vector, Burlingame, CA, USA) on phosphorylation of PKR and its substrate eIF2α were determined under cell-free conditions employing a translationally-active HeLa cell lysate (Thermo Scientific, Wilmington, DE, USA). This proprietary lysate contains the necessary cellular components for protein synthesis including ribosomes and tRNAs as well as initiation, elongation and termination factors, but is devoid of cell membrane, nuclei, mitochondria, DNA and mRNA. For kinase assays, incubation mixtures (50 µL) contained 50 µg HeLa lysate, 15 mM HEPES-KOH, pH 7.4, 5 mM Mg(OAc), 20 mM KCl, 1 mM DTT, 1 × Halt protease phosphatase inhibitor cocktail (Thermo Scientific, Wilmington, DE, USA), 1 mM EDTA and 0.1 mM ATP. Selected concentrations of ribotoxins were added to the kinase assay mixture. Poly (IC) (100 ng/mL), a known PKR activator, was used as a positive control. After incubation at 30 °C for 20 min, reactions were terminated by addition of 10 µL of 6× Laemmli SDS sample buffer (Bio-Rad, Hercules, CA, USA). In some studies, PKR inhibitors 2-AP (2 mM) or the imidizole-oxindole C16 (2 µM) (Calbiochem, La Jolla, CA, USA) [48] were used at concentrations consistent with those employed previously to block PKR activation in intact cells [10].

Relative PKR and eIF2α phosphorylation in the terminated assay mixtures were measured by qualitative Western analysis using mouse monoclonal anti-PKR antibody, rabbit polyclonal anti-PKR (pT451) phospho-specific antibody (Invitrogen, Carlsbad, CA, USA), mouse monoclonal anti-eIF2α or rabbit polyclonal anti-phospho-eIF2α (Ser51) antibody (Cell Signaling, Beverly, MA, USA) in conjunction with secondary IRDye 680 goat anti-rabbit and/or IRDye 800CW goat anti-mouse IgG antibodies (Li-Cor). Infrared fluorescence from these two antibody conjugates was measured simultaneously using the Li-Cor Odyssey Infrared Imaging System.

### 4.4. Assessment rRNA Integrity

The potential for ribotoxins to induce rRNA fragmentation in ATP-containing HeLa lysates under the conditions described above was assessed by capillary electrophoresis using an Agilent 2100 Bioanalyzer with a Nano Chip (Agilent, Santa Clara, CA, USA) as previously described [45].

### 4.5. RNA Immunoprecipitation (RNA-IP) of rRNA-PKR Complexes

RNA-IP was performed using an RNA ChIP Kit from Active Motif (Carlsbad, CA, USA), per manufacturer’s instructions with modifications. Briefly, HeLa lysates (containing PKR and ribosomes) in kinase buffer supplemented with 0.1 mM ATP were incubated with or without DON (250 ng/mL) at 30 °C for 20 min. Reaction mixtures were fixed in 1% (*v*/*v*) formaldehyde for 10 min at 25 °C. After adding glycine stop-fix solution for 5 min at 25 °C, the fixed ribosome-PKR complexes were pelleted on a sucrose cushion (10% [*w*/*v*] sucrose, 15 mM Tris pH7.4, 50 mM KCl, 10 mM MgCl_2_, 1 mM DTT, 1× Halt protease and phosphatase inhibitor cocktail, 1 mM EDTA) by ultracentrifugation at 200,000× *g* for 3 h at 4 °C. Pellets were then suspended in shearing buffer (provided in the kit) and sheared by a 60 Sonic Dismembrator (Fisher Scientific, Pittsburg, PA, USA) under preoptimized shearing conditions (40% maximum power, 5 pulses of 20 s with a 30 s rest on ice between each pulse). Resultant RNA from this treatment was primarily between 100 and 500 nucleotides based on capillary electrophoresis.

Immunoprecipitation was carried out by adding rabbit polyclonal PKR (K-17) antibody or isotype matched control antibody (Santa Cruz, CA, USA) (2 µg/100 µL IP reaction) to a mixture containing sheared rRNA-PKR complexes, protein G magnetic beads, RNase inhibitor and protease inhibitor cocktail provided in the Activ Motif Kit. After washing with RNA-ChIP buffer overnight at 4 °C, IP complexes were released with elution buffer. Efficacy of this immunoprecipitation and specificity of PKR (K-17) antibody on sheared RNA was verified in preliminary experiments by demonstrating efficacious pull-down of PKR but not RPS6 or RPL7 which are markers of 40S and 60S subunits, respectively. Resultant PKR-bound rRNA fragments were then digested by adding 5 µL proteinase K (provided in kit) at 42 °C for 1 h and cross-linking reversed by incubation at 65 °C for 1.5 h. The resulting rRNA fragments were then extracted with TRI reagent solution from Ambion (Carlsbad, CA, USA) and BCP (1-bromo-3-chloropropane) phase separation reagent (Molecular Research Center, Cincinnati, OH, USA) according to the manufacturer’s instructions. rRNA concentrations were measured using a NanoDrop Spectrophotometer (ThermoFisher, Wilmington, DE, USA).

### 4.6. Cloning and Sequencing of PKR-Associated rRNA Fragments

Purified immunoprecipitated rRNAs were cloned at the Genomics Core of Michigan State University Research Technology Support Facility. Briefly, the rRNA fragments were first treated with Antarctic Phosphatase (New England Biolabs, Ipswich, MA, USA) and subsequently T4 polynucleotide kinase (Illumina Inc., San Diego, CA, USA). Specific 5' (5'-GUUCAGAGUUCUACAGUCCCACACGAUC-3') and 3' (5rApp/ATCTCGTATGCCGTCTTCTGCTTG/3ddC) adapters were then ligated onto RNA fragments, and the resulting molecules were reverse transcribed using a primer complementary to the 3' adapter (CAAGCAGAAGACGGCATACGA). cDNA products were amplified by PCR using forward primer: GAAGCAGAAGACGGCATACGA and reverse primer: AATGATACCGCGACCACCGACAGGTTCAGAGTTCTACAGTCCGA. Resultant double-stranded DNAs were cloned into pCR2.1 vector (Invitrogen, Carlsbad, CA, USA) and transformed into chemically competent One-Shot TOP10 cells (Invitrogen, Carlsbad, CA, USA). After shaking at 37 °C, 250 rpm, for 1 h, the cells were spread on LB plate with 100 µg/mL ampicillin and cultured at 37 °C overnight. Transformed single colonies were selected and sequenced using the M13 forward primer and Applied Biosystems BigDye terminator v3.1 chemistry on an Applied Biosystems 3730xl sequencer (Life Technologies, Grand Island, NY, USA). Resulting sequences were then aligned to human 18S (NCBI Reference Sequence: NR_003286.2) and 28S (NCBI Reference Sequence: NR_003287.2) ribosomal RNA genes.

### 4.7. Prediction of the Secondary Structures of PKR-Bound rRNA Sequences

PKR-bound rRNA sequences were mapped to secondary structures previously described for human 18S and 28S rRNAs [49,50]. Exact nucleotide positions were updated using aforementioned 18S (NR_003286.2) and 28S (NR_003287.2) rRNA sequences. Selected rRNA sequences were aligned to 18S and 28S molecules using PseudoViewer web application V3.0 (http://pseudoviewer.inha.ac.kr/, last accessed 12/11/2014, Inha University, Inchon, Korea). In addition, secondary structures of the selected isolated PKR-associated rRNA sequences were predicted using the online software PknotsRG with minimum free energy (http://bibiserv.techfak.uni-bielefeld.de/pknotsrg/submission.html, Bielefeld University, Bielefeld, Germany) and then depicted with PseudoViewer.

### 4.8. RNase Protection Assay

RNase protection assays were performed using RPA III™ Ribonuclease Protection Assay Kit (Invitrogen, Carlsbad, CA, USA) following the manufacturer’s instructions. Briefly, 500 ng PKR-specific immunoprecipitated RNA and 500 pg ^32^P labeled probe were precipitated with 70% (*v*/*v*) ethanol, resuspended in 10 µL hybridization buffer, denatured at 95 °C for 5 min and incubated overnight at 42 °C. Then 150 µL RNase A/T1 solution (1:100 dilution in RNase digestion III buffer) was added and incubated at 37 °C for 30 min followed by adding 225 µL RNase Inactivation/precipitation III solution. Tubes were vortexed, centrifuged briefly and stored at −80 °C for 30 min. Precipitated RNA was collected by centrifugation at 19,000× *g* for 15 min, resuspended in 10 µL RNA loading buffer and denatured at 95 °C for 5 min. The resultant RNA was separated on 15% urea-PAGE. The gels were assembled with Hyblot autoradiography film (Denville, Metuchen, NJ, USA) into an X-ray exposure cassette and the film was developed after 24 h.

## 5. Conclusions

Taken together, the findings described here are most consistent with a sentinel model for PKR-mediated RSR initiation (Figure 10). Specifically, we hypothesize that strategic basal positioning of monomeric PKR molecules throughout the 18S and 28S rRNAs would enable them to rapidly respond to subtle ribotoxin-induced alterations in secondary and/or tertiary rRNA structure, in the absence of binding of additional molecules of the kinase. This proposed role for PKR as a ribosome guardian raises some intriguing new questions concerning the spatial-dynamics of putative ribotoxin-induced rRNA conformational changes as well as how these relate to PKR monomeric/dimeric states, autophosphorylation and downstream mobilization of signaling molecules. Furthermore, in addition to specific rRNA binding, the potential exists for PKR to interact with ribosomal proteins in both a specific and non-specific manner. The importance of such interactions cannot be discounted and should be addressed in future investigations. While outside the scope of the present study, such questions could be addressed using the novel procedures described here for stoichiometry, cell-free reconstitution of ribotoxin-induced PKR activation and RNA-IP in conjunction with existing methods for affinity labeling and structural modeling. In addition to ribotoxins, it will be of considerable interest to determine the role of PKR in sensing alterations in rRNA structure by ultraviolet radiation [51] or by chemical agents that cause oxidation, chlorination, nitration, and alkylation [52].
